# Mapping the Structure of Food Waste Management Research: A Co-Keyword Analysis

**DOI:** 10.3390/ijerph17134798

**Published:** 2020-07-03

**Authors:** Iwona Gorzeń-Mitka, Beata Bilska, Marzena Tomaszewska, Danuta Kołożyn-Krajewska

**Affiliations:** 1 Faculty of Management, Czestochowa University of Technology, 42-200 Czestochowa, Poland; iwona.gorzen-mitka@wz.pcz.pl; 2 Department of Food Gastronomy and Food Hygiene, Institute of Human Nutrition Sciences, Warsaw University of Life Sciences, 02-776 Warsaw, Poland; marzena_tomaszewska@sggw.edu.pl (M.T.); danuta_kolozyn_krajewska@sggw.edu.pl (D.K.-K.)

**Keywords:** food waste, food loss, management, co-occurrence analysis, network analysis

## Abstract

Food loss and waste represent a global problem in the ethical, social, environmental, and economic contexts. The aim of this article is to identify leading concepts in studies on food loss and waste in management research by network analysis of the co-occurrence of keywords, via mapping of knowledge domains, a method used in bibliometrics. We analyzed 2202 records from the Scopus database on food waste management with the aid of the VOSviewer software tool. In particular, keyword co-occurrence analysis was adopted to visually explore knowledge bases, topic distribution, and research fronts in the field of food waste management research. Ten representative areas were found concentrated in main keywords, namely, food waste, waste management, food, anaerobic digestion, waste disposal, recycling, waste treatment, municipal solid waste, solid waste, and refuse disposal.

## 1. Introduction

Due to the complexity of the food chain–that is, its multistage, complex organizational structure–the process of rational food flow management represents a significant challenge. As a result of errors, the rise in the volume of goods available to purchase, and the extension of distribution and logistics channels, the scale of food loss and food waste (FLW) is increasing globally. At the agricultural production stage, losses may arise due to, e.g., overproduction or grading because of quality standards. In food production and distribution, losses may result from excess stock. At the stage of consumers, losses may occur due to, e.g., consumer preferences or the preparation of oversized meals [1,2].

The aim of this study is to identify leading concepts in food waste management research through an academic literature search and bibliometric analysis that employed keyword co-occurrence analysis.

As Martin-Rios et al. [3] indicated, food waste is an ecological, economic, and social problem. Reducing food waste plays an important role in global food security [4]. Food waste has a high carbon, water, and ecological footprint. The economic impact of food losses and waste on the food system depends on the level: consumers and businesses that spend large portions of their budgets on foods that will not be consumed (micro level) reduce the financial resources available to be used for investment in other areas (macro level) [5].

There are currently no universally accepted definitions of the terms “food loss” (FL) and “food waste” (FW), either applied in a European or national legal framework, or found in publications. As the FAO [6] definition indicates, food loss is the decrease in the quantity or quality of food resulting from decisions and actions by food suppliers in the chain. On the other hand, food waste refers to the decrease in the quantity or quality of food resulting from decisions and actions by retailers, food service establishments, and consumers.

According to the project “Technology options for feeding 10 billion people—Options for Cutting Food Waste”, FL means food produced for human consumption that for various reasons falls out of the supply chain [7]. The EU Fusions project group established at the European Commission only uses the term “waste”, which refers to both edible and non-edible parts of food, such as bones and husks [8].

The High Level Panel of Experts (HLPE) defined FL as “A decrease in the food chain, excluding consumers, in the mass of food that was originally intended for human consumption, regardless of the cause”. HLPE defined FW as “food appropriate for human consumption being discarded or left to spoil at consumer level—regardless of the cause” [5].

Given that the majority of the definitions of the terms “food loss” and “food waste” are similar and place an emphasis on reducing the amount of food intended for human consumption, this understanding of the term was adopted for this article.

The FAO estimated FLW at one-third of the total food produced [9]. The European Commission estimated that between one-third and one-half of all food produced in the world is lost or wasted. Eurostat estimated, based on data provided by the EU-27, that in 2006 approximately 89 million tons of food waste were generated [10].

Inefficient management of raw materials and food products, which leads to a given batch of food no longer being suitable for human consumption, is at the same time a waste of the human labor input previously invested in its production and an irreversible consumption of natural resources. It also incurs financial costs, which are estimated at about USD $936 billion [11]. In addition to quantitative losses, inefficient use of food poses a threat to the environment, causes excessive consumption of natural resources (land, water, fertilizers, energy) [12], affects global warming, and thus constitutes a barrier to the sustainability of the food sector at a global scale. It is estimated that in developed countries the food system is responsible for 15–28% of total greenhouse gas emissions [13] and methane, which has a global warming potential 25 times higher than carbon dioxide [14].

According to Zhao et al. [15], FW has been assigned a key role in achieving Goal 12.3 of the United Nations Environment Programme. Currently, the management of food waste is segmented, but a holistic approach is needed [15,16]. According to Närvänen et al. [16], the change is needed at three different levels: actors, systems, and sociocultural and institutional structures. As Lipinski et al. [17] indicate, it also requires changes in technologies, practices, behavior, and policy. Fiore et al. [18] suggest that interventions should be taken by policy makers and social marketers to influence consumers’ choices related to purchasing and consuming food, such as changing their planning and shopping routines. Fiore et al. [19] note a lack of messages promoting sustainable consumption.

Analysis of the available literature regarding current research on food waste is an important source of information not only for scientists, but also for governmental organizations and policymakers. With such a data set, it is possible to identify research gaps and on this basis plan further actions and research.

Enterprises for which sustainable development has become an important element of building competitive advantage will look for solutions, both organizational and technological, that will allow them to reduce the burden on the environment and to use resources more efficiently. One element of sustainable development for enterprises in the food industry is sustainable production, which is disturbed by food losses and food waste.

The overall impact of these issues is to make food waste one of the most important global topics of concern, not only for organizations engaged in food markets and for food policy [20], but also for scientists [21].

The results of food waste management research have been presented in the form of a knowledge map [22,23]. Mapping of knowledge domains (MKDs) and creating knowledge maps is an important research technique in bibliometrics. It provides a visual perspective for researchers and helps them to clearly understand the general situations of particular research fields and identify, e.g., new research trends [24,25]. The data for the current analysis was provided by the Scopus database, which stores the largest amount of information meeting the selection criteria adopted in this study (these criteria are indicated in the Materials and Methods section). Next, a keyword analysis was performed using the VOSviewer software, in which analysis of bibliographic data with the clustering technique is possible (specifically, the VOSviewer clustering technique) [26,27,28,29]. This allowed us to obtain a network of interactions and to identify six groups of terms (clusters) with interrelated keywords related to issues of food waste management. The main methods used in preparing this article consist of an overview of the academic literature (especially in the Scopus database) and network analysis. This procedure made it possible to identify leading research in the area of food waste management.

In this study, the following research hypotheses were adopted:

**Hypothesis 1** **(H1).** *There has been a meaningful increase in scientific studies (as measured by the number of publications) that analyze issues of food waste in the management research literature*.

**Hypothesis 2** **(H2).** *Bibliometric analysis of keywords (selected in relation to issues of food waste management) allows determination of groups (clusters) of interrelated keywords*.

**Hypothesis 3** **(H3).** *Analysis of the indicators (i.e., occurrence ratio and total link strength) in particular groups (clusters) makes it possible to identify the leading research trend or trends in the area of food waste management research*.

## 2. Materials and Methods

This article presents the results of bibliometric analysis of the studies regarding food waste and food loss in management research. Food waste management includes the activities and actions required to manage food waste from its inception to its final disposal. We understand food waste management as any aspects of management related to the issue of food waste. Food waste management is generally perceived as a complex issue without a single universal solution. Management of food waste is an active research area that has developed significantly in recent years. Our specific approach makes it possible to identify trends in a wide stream of research as management problems in the field of food waste. In addition, since the study concerns the analysis of the words contained in the title, abstract, and keywords of selected publications (which are universally recognized as the key elements of a scientific publication when searching for information on a topic of interest), we believe that the authors’ own choices in this respect (i.e., use of the word “management” in any of the aforementioned parts of the scientific publication) is meaningful to the study of issues regarding management. Our sole focus on food waste research related to “management” delimited the literature review, but we did not limit our research to management issues in a specific area (e.g., business administration or supply chain management).

Food waste management is becoming increasingly important. Any initiatives taken by governmental and non-governmental organizations to reduce food loss and food waste in fact require a management process. Food waste is a multidimensional problem, but we decided to focus on management. This limitation was deliberate, because we wanted to focus on a new approach to food waste management research. We found that by applying this criterion, we obtained 2202 results, which proved sufficient for further analysis. The added value of our research lies in its multidisciplinary approach in combination with the methodological approach (we used specific mapping and clustering techniques), while other studies on research have focused on a silo approach, investigating one selected area of management. This allowed us to identify new approaches to food waste management research. 

The procedure in food waste management research using the VOSviewer software was as follows:

1. Identification of publications in the selected database of scientific publications (in this case, SCOPUS) based on the adopted criteria (in our case, “food waste” AND “management” until 2019, which resulted in 2202 publications).
2. Creating and downloading the bibliographical data of the selected publications from the SCOPUS database in .csv or .ris format.
3. Choosing the option in VOSviewer allowing creation of a map based on bibliographical data following the software’s recommendations (i.e., load the data downloaded from SCOPUS, select the option to research keyword cooccurrence).
4. Verification of the terms selected by the software, i.e., filtering out “interference” such as general noun words (e.g., “study”, “implications”, “introduction”) and articles (e.g., “the”, “a”, “an”), modal words (e.g., “can”, “will”, “should”), pronouns (e.g., “I”, “we”, “they”), and publishing-related words (e.g., “SCOPUS”, “Palgrave”, “copyright”).
5. Generation of a map of keyword relationships and analysis of it using the “items” and “analysis” options.

The resulting connection network is quite compact and is characterized by numerous connections in selected parts of the map (occurrence ratio, OR—the number of co-occurrences of two keywords measuring the number of publications in which both keywords occur together in the title, abstract, or keyword list; and link strength, TLS—the cumulative strength of the links of an item with other items [26]). As van Eck and Waltman [26] indicate, the construction of the keywords co-occurrence matrix is the basis of conducting cluster analysis. Counting the number of times that any two keywords appear in the same documents, n keywords can build an n_x_n co-occurrence matrix, which defines the similarity matrix S:

**S = (s_ij_), where s_ij_ ≥ 0 is a similarity measure defined below and s_ji_ =s_ij_; i,j∈ {1, 2,⋯, n}** (1)

VOSviewer uses the association strength method to calculate the similarity s_ij_ between the objects i and j in a map as:

s_ij_ = c_ij_/W_i_x W_j_, where s_ij_ denotes the similarity between the objects i and j; c_ij_ denotes the co-occurrence times of the objects i and j; and W_i_ and W_j_ denote the number of occurrences of the objects i and j, respectively.

Since both the technical information and applications of VOSviewer, and the VOS mapping and clustering techniques, have been discussed in detail in numerous publications by the developers on the software itself e.g., [24,26,27,28,29,30], as well as by other researchers using it (an extensive list of over 500 publications can be found online at https://www.vosviewer.com/publications and elsewhere), we have decided not to reproduce these in this work.

**Figure 1:**
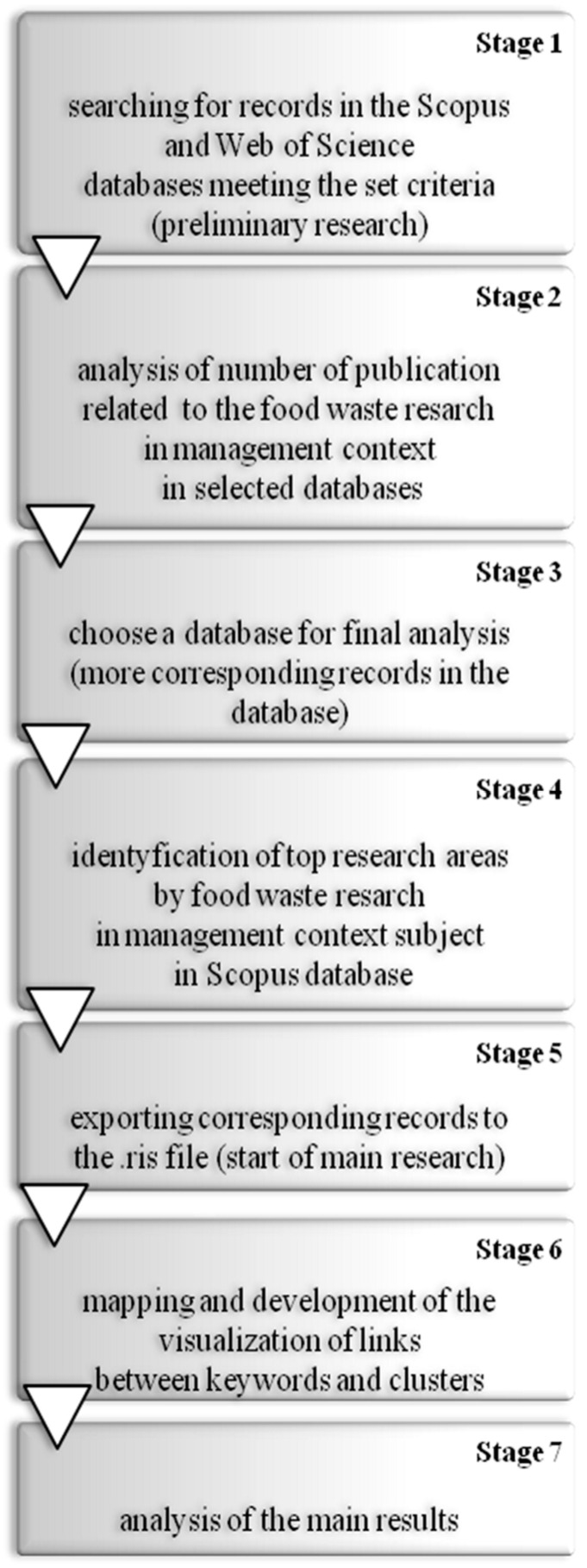
Co-occurrence analysis of key trends in food waste management research. Stage 1 = “set criteria” means the search for terms “food waste” and management in the full SCOPUS database in the topic (i.e., article title, abstract, and keywords).

In the first stage of this research, the authors used two databases, the Web of Science (WoS) and Scopus, to meet the set criteria (Figure 1). In the second stage, analysis of the number of publications related to food waste management research in selected databases was conducted. This allowed choosing a database for final analysis (more corresponding records in the database) and confirmation of hypothesis H1 (stage 3). In the next stages (4 and 5), we identified in the chosen database all publications, according to the set criteria adopted (“food waste” and management in the topic—title, abstract, and keywords) relating to food waste management research, and exported the relevant bibliometric information of all selected records to a .ris file to start the main part of the study. In the last stages (6 and 7), we mapped and developed the visualization of links between keywords in the VOSviewer software and conducted the analysis of the identified clusters (confirmation of hypothesis H2 and H3).

## 3. Results and Discussion

Research activity on FLW in management research was assessed by the number of publications. This allowed us to verify the H1 hypothesis that there has been a meaningful increase in scientific research (as measured by the number of publications) on issues of food waste management. By plotting the quantity of literature over time and conducting multivariate statistical analysis, one can understand the level of research and the future development trend in a certain field.

**Figure 2:**
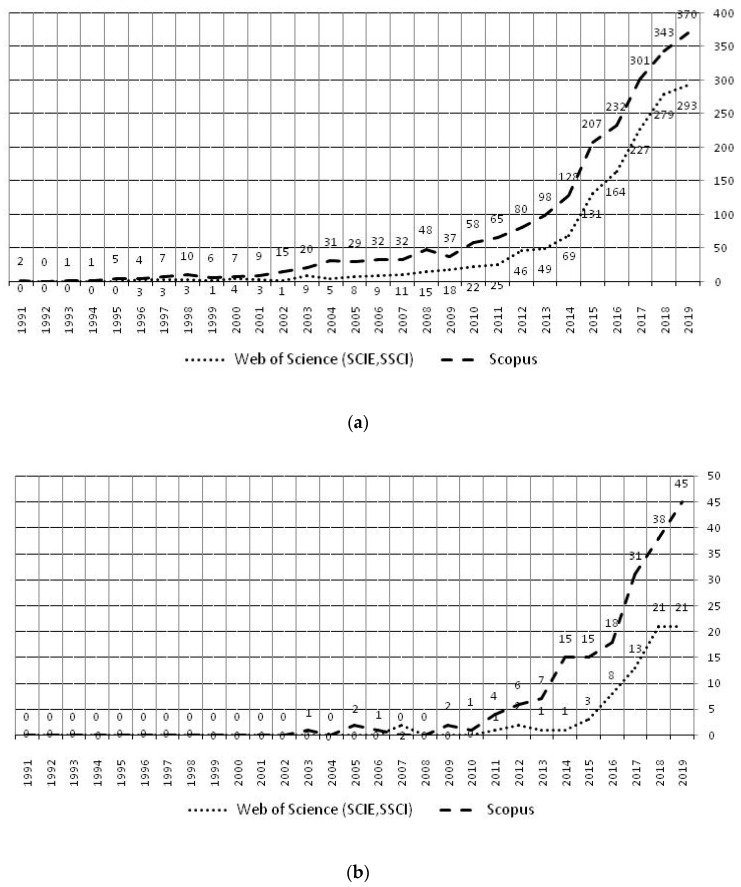
Quantitative distribution of published articles in food waste and food loss in management research: (**a**) topic “food waste” + “management”; (**b**) “food loss” + “management” in the Scopus and Web of Science databases in 1991–2019.

In the first stage of the research, the number of publications related to the terms “food loss” and “food waste” was analyzed (Figure 2) in management research. The results regarding the occurrences of the term “food loss” in scientific publications in both databases used for the analysis show that these publications are few in number and did not exceed 50 per year, with a total number of 192 for the analyzed period in the Scopus database and only 73 in the WoS database. Moreover, the analysis of keyword groups of these publications indicates that the issues related to “food loss” are, in the majority of cases, analyzed in management research in relation to the term “food waste”. With this in mind, publications related to the topic of “food waste” + “management” were finally selected for the analysis of keyword co-occurrence. 

The breakdown of the number of publications is presented in Figure 2.

Figure 2 (second stage of research) shows that the number of publications related to the term “food waste” in “management” research (topic “food waste” + “management”) is similar in both databases, but generally more publications were collected in Scopus. 

The quantity of documents relating to food waste management research has progressed through three stages—“initial”, “primary”, and “fast-growing”—which are explained below:

- Initial stage (1991–2000)—from the first article regarding food waste management studies published in 1991 to the 2000s, there were few related research results in this field, and the maximum annual number of published papers was only 10 (Scopus database), which means a complete document system had not yet been formed. (The first publication indexed in the WoS database is Wilson and Huang [31]. The first publication indexed in the Scopus database is Eckenfelder [32]).
- Primary stage (2001–2013)—the number of documents in this stage started to rise meaningfully, with an average annual growth of eight articles. It can be considered that the research field of food waste management research was initially formed and growing systematically during this period.
- Fast growing stage (2014–2019)—the number of publications grew annually by 40 articles on average. This indicates that work on food waste management research grew intensively and entered a phase of rapid development.

It can be seen in Figure 2a that during the entire period under consideration, more articles on the topic of food waste management were indexed in the Scopus database. The H1 hypothesis was thus verified. Moreover, as a result of the initial investigation of databases and evaluation of the number of publications (a database was considered ineligible for this study if there was too low a number of aspects of interest to the authors), as well as the number of duplicated articles (the WoS database), the Scopus database was identified as representative. Therefore, the Scopus database was selected for further analysis (third stage of research).

In next (fourth) stage of research, the main areas of investigation related to the topic of food waste management research were identified. The analysis was concluded in relation to this criterion, which involved the number of scientific articles in the top ten subject areas.

**Figure 3:**
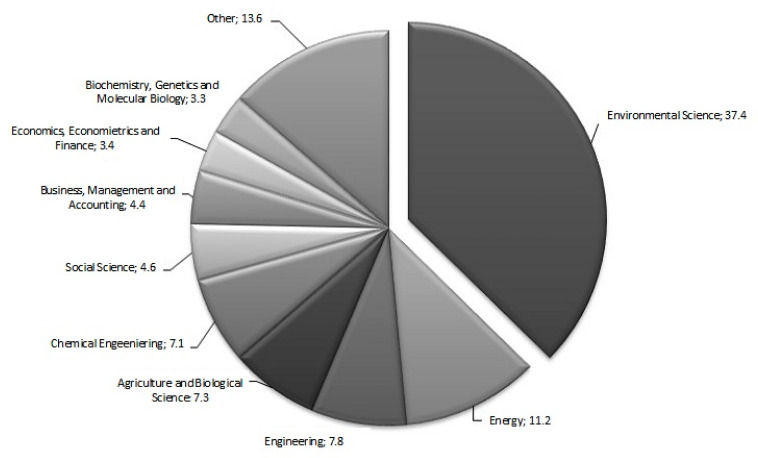
The main research areas in the subject of food waste management (%)—the food waste and management terms, searched in the full SCOPUS database in the topic (i.e., article title, abstract, and keywords); Source: own processing, data extracted from the Scopus database.

It can be seen in Figure 3 (fourth stage of research) that the nature of food waste management research by subject area has been quite concentrated. Researchers have worked in fields such as Environmental Science (37.4%) and Energy (11.2%).

Interesting information is also provided by the observation of the spatial distribution of the study authors (by country). This indicates that the authors who have investigated issues of food waste management in their publications are most often from the United States (362 articles—16.4%), China (268 articles—12.2%), the United Kingdom (214 articles—9.7%), and Italy (138 articles—6.3%), which collectively represent almost 40% of the total number of all studies in this field.

**Figure 4:**
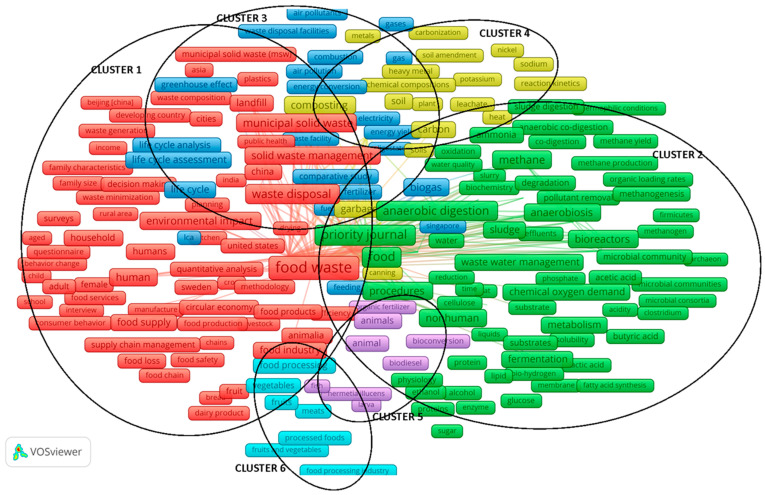
Keyword co-occurrence network of food waste studies in management studies.

Keyword co-occurrence analysis is used to analyze the link strength between the co-occurrence of keywords by studying the relation of their co-occurrence in a large number of documents (in this case, 2202 research papers from the Scopus database regarding management). Its main aim is to describe the internal relationship and structure, as well as to reveal the research fronts of a particular academic discipline. Research front here refers to, inter alia, basic problems, as well as the rise or unexpected emergence of theoretical trends and new topics. The results of the keyword co-occurrence analysis of food waste management research (sixth stage of research) with the VOSviewer software are shown in Figure 4. Data selection and research procedures using the VOSviewer application were adapted from studies by van Eck and Waltman [26], Gudanowska [25], and Xin et al. [22]. The analysis of the co-occurrence of keywords was performed with the use of VOSviewer software following van Eck and Waltman [26] and Xin et al. [22]. The analysis was based on the keywords provided by the authors of the publications. As Xin et al. [22] indicate, the keywords are an important indicator in bibliometrics. Keyword co-occurrence analysis is based on the statistics of the number of times a pair of keywords is cited in the same document; 13,137 keywords were identified in the course of the analysis. To present a clear visualization, this paper focuses on those expressions that appeared at least 10 times in a group of selected publications (this limited the group of keywords to 891).

**Figure 5:**
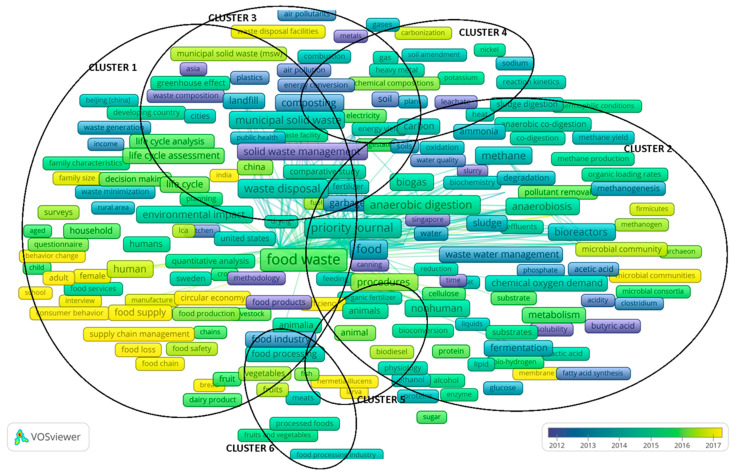
Keyword co-occurrence network of food waste studies in management studies detected over time.

Figure 4 presents the resulting map (a whole map of co-occurrence keywords) and Figure 5 presents the resulting maps of clusters detected over time. In Figure 4, it can be seen that the topics of food waste in management studies form six clusters. The map includes the most frequently occurring keywords. The size (height of the element on the map) of the nodes [26] representing each of the keywords, as well as the font size in which the name of a given node is written, correspond to the frequency of the occurrence of a given term.

The distance between items in the visualization approximately indicates their relatedness in the co-occurrence network. The distance is understood as the interval between the nodes. In the distance-based approach, the nodes in a bibliometric network are positioned in such a way that the distance between two nodes approximately indicates the relatedness of the nodes [29]. Items are understood as objects of interest (e.g., publications, researchers, keywords, authors [24]). In general, the closer the two items are located to each other, the stronger their relatedness in terms of occurrence links in the analyzed group of publications [24]. This allowed us to verify the H2 hypothesis that bibliometric analysis of keywords (selected in relation to issues of food waste management) allows determination of groups (clusters) of interrelated keywords.

Additionally, the resulting connection network is quite compact and is characterized by numerous connections in selected parts of the map (occurrence ratio—OR, and total link strength—TLS).

The top 10 keywords with the highest occurrence ratio (OR) and total link strength (TLS) are as follows: food waste (OR = 1293; TLS = 26,847), waste management (OR = 1180; TLS = 24,101), food (OR = 495; TLS = 13,706), anaerobic digestion (OR = 465; TLS = 12,000), waste disposal (OR = 418; TLS = 10,759), recycling (OR = 331; TLS = 7249), waste treatment (OR = 328; TLS = 8540), municipal solid waste (OR = 304; TLS = 6934), solid waste (OR = 302; TLS = 8377), and refuse disposal (OR = 294; TLS = 8933). Details on the main keywords and their characteristics by co-occurrence and total link strength related to this map are presented in the analysis of individual clusters.

Cluster 1 (red) shows keywords of coexistence (10 keywords with the highest number of occurrences) namely, food waste, waste management, waste disposal, recycling, municipal solid waste, human, solid waste management, anaerobiosis, landfill, sustainable development. 

The first cluster classified in VOSviewer (Cluster 1, Figure 4 and Figure 5; Table 1) is a group of issues related to food waste, waste management, and sustainable development, among others. This cluster is the most numerous among all of those classified, due to the number of links and their strength. The leading keyword in Cluster 1 is food waste. As the depicted links indicate, the issue of food waste is primarily considered in the context of sustainable development. The leading group of studies concerns the analysis of food waste due to issues of waste management, especially disposal, storage, and recycling (e.g., Iacovidou et al. [33], Paritosh et al. [34]). The research focuses on the issues of municipal waste and solid waste management. Examples of this research are Peng et al. [35], Ng et al. [36]. The problems observed in Cluster 1 are reflected both in the latest scientific literature, as well as in economic and political recommendations. For example, solid waste management is one of the key services every city government must provide with widely variable service levels, costs, and environmental impacts. One researcher who emphasizes this is Parry [37], who analyzed selected sustainable food waste management alternatives (economic, environmental, social, and operational impacts), e.g., de-centralized composting, for a hypothetical community of 100,000 residents. In turn, Edwards et al. [38] proposed in their study a specific approach determining the efficiency of a system to turn waste into a valuable resource. On the map of trends, food waste is clearly shifted toward the center of the map, which indicates its numerous stronger connections with a large number of other issues. In this cluster, we can find few relatively new elements (see Figure 5).

Due to the links between the keywords over time, the analysis of the cluster shows that the problem of food waste is considered in the context of its environmental impact (the link between elements is quite strong). This kind of research is presented by Edwards et al. [38] and Koido et al. [39]. The most recent research trends in this area chiefly concern analyses in the context of supply chain management, problems related to food loss, food waste management, and changes in consumer behavior and consumption behavior. Examples of this are Pellegrini et al. [40], who analyze the factors affecting consumer food waste behavior at a household level, and Bhatti et al. [41], who investigate the factors that affect young consumers’ food waste behavior in the context of a developing country. Importantly, the most recent research concerns analyses in the context of the circular economy (this is discussed, e.g., in the study by Loizia et al. [42]). It can be said that food waste in management research (especially in supply chain management) in these areas is a new trend. This is confirmed by research by Zhao et al. [43], in which the authors also point to value chain models to reduce food waste and forecasting food waste as an area of current research inquiries.

Cluster 2 (green) shows keywords of coexistence (10 keywords with the highest number of occurrences), namely, food, anaerobic digestion, methane, controlled study, procedures, nonhuman, bioreactors, environmental impact, sewage, bioreactor. 

The second cluster classified in VOSviewer (Cluster 2, Figure 4 and Figure 5, Table 2) is a group of issues related to wastewater management in the anaerobic co-digestion context (digestion, fermentation, growth, metabolism, process, reactor, treatments, among others). The most frequent and the most interrelated components in the cluster were food and anaerobic digestion. These components were presented, for example, by Loizia et al. [42], Nguyen et al. [44], and Singlitico et al. [45].

In this cluster, we can find relatively new elements (see Figure 5). This may indicate that research on, e.g., food waste in the anaerobic co-digestion context is at a rapid growth stage (the link between elements is quite strong). This cluster, like the first, is relatively more numerous, due to the number of links and their strength, among all those classified. The leading keywords in Cluster 2 are food and anaerobic digestion. As the illustrated links indicate, these issues are considered primarily in the context of wastewater management. This issue has been studied, for example, in work by Maalouf and El-Fadel [46]. This study explores the economic dimension of introducing a food waste disposer (FWD) policy in the context of its implications for solid waste and wastewater management. As the authors indicate [46], the sensitivity analyses on processes with a wide range of costs showed an equivalent economic impact, thus emphasizing that the viability of an FWD policy although the variation in the cost of sludge management exhibited a meaningful impact on savings.

The leading group of studies concerns analyses related to anaerobic co-digestion, particularly the role of microbial methanogenic bacterium in pollutant removal. On the trend map, food waste is clearly shifted toward the center of the cluster, which indicates its numerous stronger connections with a large number of other issues. In this cluster, we can find few relatively new elements (see Figure 5).

This is confirmed by the cluster analysis due to the links between the keywords over time (the link between elements is quite strong). The most recent research trends in this area chiefly concern analyses in the context of issues in wastewater management in relation to such topics as biofuels, methanogenesis, and pollutant removal (biodiesel, methanogenesis).

It can be said that food waste in management research (especially in wastewater management) in these areas is a new trend. Food waste is increasingly viewed as a resource that should be diverted from landfills. For example, Beckeret al. [47] used life cycle assessment to compare co-management of food waste and domestic wastewater using an anaerobic membrane bioreactor versus conventional activated sludge and high-rate activated sludge with three disposal options for food waste: landfilling, anaerobic digestion, and composting.

Cluster 3 (blue) shows keywords of coexistence (10 keywords with the highest number of occurrences), namely, composting, anaerobic growth, chemical oxygen demand, incineration, chemistry, greenhouse gases, life cycle analysis, supply chains, waste incineration, land fill. 

The third cluster (Cluster 3, Figure 4 and Figure 5; Table 3) is generally associated with the chemical processes related to composting, greenhouse gases, and waste incineration. This cluster is also relatively numerous, but in comparison to Clusters 1 and 2, it groups just over 100 keywords. The leading keywords in the third cluster are composting and issues concerning anaerobic processes (including co-digestion, fermentation, metabolism, and other anaerobic treatments). These issues are a continuation of the trends outlined in Cluster 2. As the depicted links indicate, the issue of composting is considered primarily in the context of municipal solid waste management. The leading research group also concerns analysis in the area of waste management, in particular, problems of waste disposal facilities, for example, Iacovidou et al. [33]. The research focuses on the problems of municipal waste and solid waste management. On the trend map, municipal solid waste is clearly shifted toward the center of the cluster, which indicates its numerous stronger connections with a large number of other issues. In this cluster, we can find few relatively new elements (see Figure 5).

Cluster analysis due to keyword linkages over time shows that the issue of municipal solid waste is considered in the context of its link to the greenhouse effect and electricity (the link between elements is quite strong). The keyword groups observed in this cluster develop the research areas indicated in the previous clusters, further elaborating these issues. For example, identification of the decisive factors for greenhouse gas emissions in comparative life cycle assessments of food waste management was made by Bernstad et al. [48] and Bernstad et al. [49].

It can be said that research in these areas presents a grounded stable trend, but municipal solid waste in the management research (especially the greenhouse effect) is a relatively new trend.

Cluster 4 (yellow) shows keywords of coexistence (10 keywords with the highest number of occurrences), namely, waste treatment, solid waste, refuse disposal, waste, biogas, organic waste, carbon, garbage, humans, wastes. 

The fourth cluster classified (Cluster 4, Figure 4 and Figure 5, Table 4) is a group of issues associated with waste treatment in the context of solid waste, garbage (especially refuse disposal), biogas, organic waste, and carbonization processes, among others. This cluster is also relatively numerous, but in comparison to Clusters 1 and 2, it groups just over 100 keywords. The leading keyword in the fourth cluster is waste treatment. These issues are a continuation of the trends outlined in Clusters 2 and 3. On the trend map, the keywords “carbon” and “soil” are clearly shifted toward the center of the cluster, which indicates their numerous stronger connections with a large number of other issues. In this cluster, we can find relatively few new elements (see Figure 5).

Cluster analysis of keyword linkages over time shows that the issue of municipal solid waste is considered in the context of its link to the carbonization process (the link between elements is quite strong). This trend is, for example, outlined in the studies by Eriksson et al. [50] and Eriksson and Spångberg [51] examining the carbon footprint and energy use of food waste management options for fresh fruit and vegetables in supermarkets, or in studies by Maaloufand El-Fadel [52], who analyze the carbon footprint of integrated waste management systems with implications for food waste diversion into wastewater streams.

It can be said that carbonization issues (especially in waste treatment) represent a new research trend.

Cluster 5 (violet) shows keywords of coexistence (10 keywords with the highest number of occurrences) namely, animals, waste composition, chemical water pollutants, heating, consumption behavior, pollution, biological oxygen demand analysis, renewable energy resources, effluent treatment, emission. 

The last two clusters classified are relatively low in number relative to Clusters 1–4. The topics taken up in the fifth cluster (Cluster 5, Figure 4 and Figure 5; Table 5) are focused on research regarding animals. The location of Cluster 5 is clearly shifted towards the center of the whole map of keyword co-occurrence, which indicates it having some quite strong connections with other issues of food waste. Additionally, from a food waste perspective, this research is at a rather early stage (Figure 5). This research is presented by Shahariar and Rooney [53]. Despite the numerically small group of keywords, their inclusion in the clustering technique indicates their close connection. The emergence of the issue of food waste in the research of animals is signaled by research of Salemdeebi et al. [54], who conducted a comparative analysis of food waste management options in relation to the environmental and health impacts of using food waste as animal feed. 

Cluster 6 (light blue) shows keywords of coexistence (10 keywords with the highest number of occurrences), namely, food processing, industrial waste, unclassified drug, meat, physical chemistry, developing world, waste to energy, volatile organic compound, resource recovery, natural resources management. 

Finally, the sixth classified cluster (Cluster 6, Figure 4 and Figure 5; Table 6) is a group of issues associated with the general aspects of natural resources management, particularly in food processing, industrial waste, and waste in the energy context, among others. Cluster 6 is clearly shifted out of the center of the whole map of keywords co-occurrence, which indicates it having some, albeit weaker, connections with other issues.

Analysis of changes in keyword linkages over time shows that the most recent research in the area of food waste regards food processing (especially in the fruit and vegetable industry). This research from the food waste management perspective is at a rather early stage (e.g., Martin-Rios et al. [3], Otles et al. [55], Ounsaneha et al. [56], and Thamagasorn and Pharino [57]). It can be said that research in these areas is a quite new trend.

Cluster trends identified in this cluster can be found, among others, in research by Kosseva [58] regarding food waste management techniques and processing technologies, or by Garcia-Garcia et al. [59], who describe a novel decision-support tool to enable food manufacturers to evaluate a range of waste management options and identify the most sustainable solution.

The bibliometric analysis of the co-occurrence of keywords in research on food waste in the management research indicates research gaps resulting from current trends in management science. This allowed us to verify the H3 hypothesis that analysis of the indicators (i.e., occurrence ratio and total link strength) in particular groups (clusters) makes it possible to identify the leading research trend or trends in food waste management research.

## 4. Conclusions

The growth of the number of articles indicates that food waste management research is developing rapidly as a field in the global academic community. The number of publications during the past five years grew annually by 40 articles on average in the Scopus database alone, compared to average annual growth of five articles per year during the period 2000–2005.

One of the main findings of our research is the identification of six large thematic clusters, which represent a research direction in the food waste management area. Based on the calculated co-occurrence networks, we discovered related key topics in in each of the six clusters. 

The first cluster is the most numerous among those classified. This cluster is a group of issues related to food waste, waste management, and sustainable development. The frontier topics in the second cluster are a group of issues related to wastewater management in the anaerobic co-digestion context. Cluster 3 is associated with composting, greenhouse gases, and waste incineration. The fourth cluster is a group of issues associated with waste treatment, solid waste, and refuse disposal. The topics taken up in the fifth cluster are focused on using food waste as animal feed. The leading keywords in the last cluster are a group of issues associated with the general aspects of natural resource management, particularly in food processing, industrial waste, and waste in the energy context.

The leading trends in research on food waste in the management research were identified, such as food waste research (especially in supply chain management), anaerobic co-digestion, waste water management, carbonization issues (especially in waste treatment), and food processing in food waste management studies.

The research conducted makes it possible to indicate the directions for further development, both on a methodological basis and from the results obtained in the clustering technique applied. The areas to which more attention should be paid in scientific research in the context of food waste are, e.g., prevention, reuse, food security.

This area undoubtedly still poses great challenges to academic researchers and managers. The complexity and multidimensionality of the variables that determine food waste management require decision makers to take a look at the role of management (especially in problems of food waste) in company processes from different perspectives. 

In the authors’ view, the results of the research presented in this study may provide a basis for further work in this area.

### 4.1. Limitations of This Study

In the authors’ view, elements of the method used in this study may be regarded as a limitation. Specifically, the study used bibliographic data from one (although the most numerous) database of scientific publications, and focused only on the study of links between keywords, while for analysis and visualization of the results, only one software package was used (although its usefulness has been confirmed in numerous scientific publications in many fields, as indicated in this work).

### 4.2. Future Research

This research aimed to identify the selected challenges in an exploratory manner. Future research could extend the research concept identified here by complementing it with dedicated areas.

Food waste management research is undoubtedly a multidimensional concept (not only in environmental science). There are many other areas in food waste that pose challenges in management research, and future studies should investigate the relationships between other dimensions of this concept.

On the basis of the methodology adopted, we suggest that further research may include the following directions:

- The adopted interdisciplinary approach to the analysis of food waste management research, which allowed identification of new research trends (that are marginally indicated in other studies adopting the silo approach, i.e., concentration on a selected management area), can be extended by including investigation of other bibliometric databases (e.g., Web of Science-Clarivate Analytics, EconBiz, EconLit, FSTA—Food Science and Technology Abstracts, and others).
- Expanding the analysis to include, e.g., co-citation and co-authorship relationships, or full-text analysis of papers, would also allow comparison of the results obtained to date in this area.
- Interesting results might also be obtained by conducting analyses using other methods or bibliometric programs.
- In turn, based on the results obtained, we suggest that further research may include future studies focusing on managerial aspects, such as seeking new ways to use the insights of supply chain research instruments and their adaptation to the needs of food waste (which is reflected in most of the identified clusters). Finally, based on this study, future research could use these findings in environmental practice (cities, etc.).

## Figures and Tables

**Table 1:** Food waste management research—analysis of results for Cluster 1.

Cluster	Number of Keywords	Keyword (Max. Co-Occurrence)	Max. Number of Co-Occurrence for Main Keyword	Keyword (Max. Total Link Strength)	Max. Total Link Strength for Main Keyword	Keywords	Co-Occurrences	Total Link Strength
**1**	316	food waste	1293	food waste	26,847	food waste	1293	26,847
						waste management	1180	24,101
						waste disposal	418	10,759
						recycling	331	7249
						municipal solid waste	304	6934
						human	252	5615
						solid waste management	205	5219
						anaerobiosis	200	6941
						landfill	180	4701
						sustainable development	178	2886
						solid wastes	174	3559
						domestic waste	168	4238
						food supply	158	2735
						sustainability	157	2307
						biodegradation	137	3631
						catering service	117	2608
						environmental management	103	2145
						agriculture	101	2233
						carbon footprint	93	2506
						ammonia	87	2745
						animalia	83	1922
						economics	81	1767
						vegetable	80	2329
						compost	71	1527
						chemical composition	63	1853
						acetic acid	62	2064
						fatty acids	59	2040
						biotechnology	57	1631
						supply chain management	56	840
						concentration (composition)	55	1740
						substrates	54	1612
						anaerobic co-digestion	54	1671
						degradation	52	1464
						environmental monitoring	52	1492
						renewable energy	51	1342
						microbiology	51	1690
						adult	50	1043
						butyric acid	50	1651

Due to the number of keywords (316) grouped in Cluster 1, the table indicates those keywords with a minimum of 50 occurrences. Source: own processing via VOSviewer program, data extracted from the Scopus database.

**Table 2:** Food waste management research—analysis of results for Cluster 2.

Cluster	Number of Keywords	Keyword (Max. Co-Occurrence)	Max. Number of Co-Occurrence for Main Keyword	Keyword (Max. Total Link Strength)	Max. Total Link Strength for Main Keyword	Keywords	Co-Occurrences	Total Link Strength
**2**	290	food	495	food	13,706	food	495	13,706
						anaerobic digestion	465	12,000
						methane	288	8615
						controlled study	281	8588
						procedures	240	7744
						nonhuman	233	6820
						bioreactors	216	7174
						environmental impact	200	4491
						sewage	196	6063
						bioreactor	186	6251
						fermentation	151	4245
						ph	150	4744
						nitrogen	147	4414
						life cycle	145	3602
						temperature	143	4096
						waste water management	139	4218
						sludge	139	4479
						food industry	137	3231
						anoxic conditions	129	4457
						metabolism	121	3946
						life cycle assessment	120	3117
						biofuels	120	4027
						wastewater treatment	115	3345
						carbon dioxide	114	3178
						biofuel	114	3743
						volatile fatty acid	113	3892
						hydrolysis	104	3273
						waste products	98	2920
						waste water	95	2950
						biomass	92	2406
						household	87	1878
						waste disposal, fluid	80	2566
						volatile fatty acids	80	2816
						sewage sludge	77	2076
						organic matter	77	2233
						phosphorus	77	2245
						biodegradation, environmental	77	2314
						concentration (parameters)	77	2557
						food wastes	72	1546
						hydrogen	71	2225
						bioremediation	67	1933
						wastewater	67	2025
						fatty acids, volatile	66	2459
						optimization	65	1700
						recovery	64	1531
						microbial community	64	2098
						sludge digestion	63	1907
						hydrogen-ion concentration	63	2183
						environmental protection	62	1390
						acidification	59	2087
						plastic	57	1459
						water	57	1703
						fruit	55	1446
						environment	54	1338
						conservation of natural resources	54	1489
						biodegradability	54	1725
						fertilizer	53	1691
						methanogenesis	53	1836
						municipal solid waste (msw)	52	1062
						waste component removal	52	1701
						cost benefit analysis	51	1244
						manures	51	1461
						quantitative analysis	50	1169

Due to the number of keywords (290) grouped in Cluster 2, the table indicates those keywords with a minimum of 50 occurrences. Source: own processing via VOSviewer program, data extracted from the Scopus database.

**Table 3:** Food waste management research—analysis of results for Cluster 3.

Cluster	Number of Keywords	Keyword (max. Co-Occurrence)	Max. Number of Co-Occurrence for Main Keyword	Keyword (Max. Total Link Strength)	Max. Total Link Strength for Main Keyword	Keywords	Co-Occurrences	Total Link Strength
**3**	118	composting	254	composting	5692	composting	254	5692
						anaerobic growth	145	5139
						chemical oxygen demand	120	4041
						incineration	119	3239
						chemistry	114	3578
						greenhouse gases	111	2739
						life cycle analysis	107	3145
						supply chains	103	1926
						waste incineration	98	2494
						land fill	93	1951
						greenhouse gas	91	2507
						life cycle assessment (lca)	89	2151
						fertilizers	87	2396
						greenhouse effect	73	2311
						gas emissions	72	1851
						manure	69	2040
						moisture	68	1751
						fatty acid	64	2269
						global warming	63	1855
						environmental impact assessment	61	1873
						vegetables	60	1542
						decision making	59	1026
						climate change	58	1283
						leaching	53	1661
						urban area	51	1342
						biofuel production	51	1512
						models, theoretical	50	1522
						composting	254	5692
						anaerobic growth	145	5139
						chemical oxygen demand	120	4041
						incineration	119	3239
						chemistry	114	3578
						greenhouse gases	111	2739
						life cycle analysis	107	3145
						supply chains	103	1926
						waste incineration	98	2494
						land fill	93	1951
						greenhouse gas	91	2507
						life cycle assessment (lca)	89	2151
						fertilizers	87	2396
						greenhouse effect	73	2311
						gas emissions	72	1851
						manure	69	2040
						moisture	68	1751
						fatty acid	64	2269
						global warming	63	1855
						environmental impact assessment	61	1873
						vegetables	60	1542
						decision making	59	1026
						climate change	58	1283
						leaching	53	1661
						urban area	51	1342
						biofuel production	51	1512
						models, theoretical	50	1522

Due to the number of keywords (118) grouped in Cluster 3, the table indicates those keywords with a minimum of 50 occurrences. Source: own processing via VOSviewer program, data extracted from the Scopus database.

**Table 4:** Food waste the management research—analysis of results for Cluster 4.

Cluster	Number of Keywords	Keyword (Max. Co-Occurrence)	Max. Number of Co-Occurrence for Main Keyword	Keyword (Max. Total Link Strength)	Max. Total Link Strength for Main Keyword	Keywords	Co-Occurrences	Total Link Strength
**4**	116	waste treatment	328	refuse disposal	8933	waste treatment	328	8540
						solid waste	302	8377
						refuse disposal	294	8933
						waste	264	7290
						biogas	254	6750
						organic waste	139	4011
						carbon	139	4024
						garbage	127	3926
						humans	108	2595
						wastes	90	2016
						soil	78	2187
						food products	71	1264
						energy recovery	68	1940
						cities	63	1822
						comparative study	60	1789

Source: own processing via VOSviewer program, data extracted from the Scopus database.

**Table 5:** Food waste management research—analysis of results for Cluster 5.

Cluster	Number of Keywords	Keyword (Max. Co-Occurrence)	Max. Number of Co-Occurrence for Main Keyword	Keyword (Max. Total Link Strength)	Max. Total Link Strength for Main Keyword	Keywords	Co-Occurrences	Total Link Strength
**5**	30	animals	118	animals	3271	animals	118	3271
						waste composition	33	687
						chemical water pollutants	33	1056
						heating	31	855
						consumption behavior	29	459
						pollution	28	530
						biological oxygen demand analysis	25	985
						renewable energy resources	19	437
						effluent treatment	19	456
						emission	18	543
						valorisation	17	319
						carboxylic acid	17	507
						microbial diversity	17	578
						bioelectric energy sources	16	499
						resource management	15	337
						sodium chloride	15	369
						fish	15	419
						fruits and vegetables	14	386
						grass	13	385
						batch process	13	422
						efficiency measurement	12	294
						flow measurement	12	298
						filtration	11	319
						apple	10	267
						membranes, artificial	10	355
						dry weight	10	367

Source: own processing via VOSviewer program, data extracted from the Scopus database.

**Table 6:** Food waste management research—analysis of results for Cluster 6.

Cluster	Number of Keywords	Keyword (Max. Co-Occurrence)	Max. Number of Co-Occurrence for Main Keyword	Keyword (Max. Total Link Strength)	Max. Total Link Strength for Main Keyword	Keywords	Co-Occurrences	Total Link Strength
**6**	21	food processing	97	industrial waste	2338	food processing	97	2180
						industrial waste	84	2338
						unclassified drug	60	1980
						meat	41	1095
						physical chemistry	41	1229
						developing world	27	602
						waste to energy	27	615
						volatile organic compound	22	747
						resource recovery	19	564
						natural resources management	18	243
						industrial waste treatment	17	422
						socioeconomics	15	353
						kitchen	14	322
						resource efficiencies	13	235
						industrial wastes	12	182
						elastomers	10	219
						aluminum	10	221
						concentration (process)	10	261
						separation technique	10	281
						enzyme	10	303

Source: own processing via VOSviewer program, data extracted from the Scopus database.
